# The severity of valvular heart disease in euthyroid individuals is associated with thyroid hormone levels but not with TSH levels

**DOI:** 10.3389/fendo.2023.1193557

**Published:** 2023-07-04

**Authors:** Pin Wang, Sen Lu, Yan Yang, Limei Liu, Guangpeng Zhou, Jieling Zhu, Diejing Niu, Yi Wang, Shaohua Wang

**Affiliations:** ^1^ Department of Endocrinology, Medical School of Southeast University, Nanjing, China; ^2^ Department of Endocrinology, Sichuan Provincial People's Hospital, University of Electronic Science and Technology of China, Chengdu, China; ^3^ Department of Intensive Care Unit, Sichuan Academy of Medical Science & Sichuan Provincial People’s Hospital, Chengdu, Sichuan, China; ^4^ Department of Nursing, Sichuan Academy of Medical Science & Sichuan Provincial People’s Hospital, Chengdu, China; ^5^ Department of Endocrinology, Affiliated ZhongDa Hospital of Southeast University, Nanjing, China

**Keywords:** thyroid hormone, valvular heart disease, euthyroid sick syndrome, NYHA grades, age

## Abstract

**Background:**

Abnormal thyroid function is a metabolic disorder and can lead to several complications, including cardiovascular diseases. In this study, we aimed to examine the relationship between clinical traits and outcomes and the thyroid hormone level of euthyroid individuals with valvular heart disease (VHD).

**Method:**

The thyroid function was evaluated in 526 euthyroid VHD patients and 155 healthy control people. As well as clinical indicators were collected and analyzed.

**Results:**

No difference in TSH levels (p>0.05) was recorded; however, fT3, TT3, and TT4 levels were lower in the euthyroid VHD patients than in healthy control(4.3 vs 4.63; 1.37 vs 1.48; 97.7 vs 102.09, respectively, all p<0.05), while the fT4 level was higher (12.91 vs 12.35, p<0.05). Moreover, all showed a continuous trend with the change of NYHA grade which does not consist of the incidence of euthyroid sick syndrome(ESS). Further analysis showed that for every 10-fold increase in BNP, fT4 increases by 83%, fT3 decreases by 30%, and TT3 decreases by 12% after being adjusted for other influencing factors. Meanwhile, adjusted fT4 was correlated with multiple worse clinical indicators, which were influenced by age.

**Conclusion:**

Thyroid hormones are widely regulated in VHD patients even with acceptable cardiac function, except for TSH level. And the adjusted fT4 is related to worse clinical indicators and outcomes which are only recorded in patients under 53 years old.

## Introduction

Thyroid hormone impacts on the body can have a positive impact on the heart and vascular system (1). The active cellular form of thyroid hormones, triiodothyronine (T3), profoundly alters cardiovascular hemodynamics through a number of mechanisms, including direct genomic effects, extra-nuclear, nongenomic effects on the ion channels, and other effects on the peripheral circulation (2, 3). By influencing tissue oxygen consumption, vascular resistance, blood volume, cardiac contractility, and heart rate, triiodothyronine can boost cardiac output in various ways (1). Faster heart rates, ejection fractions, cardiac outputs, and blood volumes in hyperthyroidism patients indicate enhanced cardiac pump performance with lower afterloads. They also have lower systemic vascular resistance and isovolumic relaxation times (1, 2). Contrary to hyperthyroidism, hypothyroidism considerably decreases cardiac preload and increases afterload, resulting in a decrease in stroke volume and cardiac output. Even in the condition’s preclinical manifestations, this is true. Adults with hypothyroidism are more likely than euthyroid people to experience heart failure, decreased cardiac function, coronary heart disease, and all-cause mortality (4–6).

In addition to the considerable effects thyroid hormones have on the circulatory system, heart diseases are distinct pathophysiological disorders that may also have an effect on the level of thyroid hormones (7). In the past, euthyroid sick syndrome (ESS) was blamed for the irregular thyroid functions in people with cardiac disorders. However, individuals with heart disorders, in particular those with valvular heart diseases unrelated to metabolic factors, have a distinct cardiac function (8). As opposed to ESS, which is controlled by a variety of peripheral thyroid hormone regulation systems, the status of thyroid hormones in this state is more complex. Numerous studies have examined the connection between thyroid hormones and heart failure, but the most widely used indicator of thyroid function was the TSH (thyroid-stimulating hormone) level, which may not be a reliable indicator of thyroid function in heart failure because TSH levels are not correlated with triiodothyronine levels (9–12). Meanwhile, it is worth to noting that several studies have identified intriguing associations between FT4 and the heart. For instance, the Penn Heart Failure Study discovered a positive correlation between atrial fibrillation and higher levels of FT4 (not FT3 or TT3) (9). Additionally, other studies have revealed higher FT4 levels are linked to an increased risk of heart failure and sudden cardiac death (13, 14). As a result, TSH may not be the most significant indicator of thyroid function in euthyroid people with valvular heart disease due to their thyroid hormone status. However, the connection between thyroid hormone levels and the clinical signs and prognosis of valvular heart disease is not yet fully understood.

Thus, studying the thyroid hormone levels in euthyroid patients with valvular heart disease and analyzing their correlation with clinical traits and outcomes are the goals of this clinical study.

## Materials and methods

### Patients

From January 2019 to June 2021, the Sichuan Provincial People’s Hospital’s Department of Endocrinology, cardiac surgery, intensive care unit, and health examination center conducted this retrospective study. It was accepted by the Sichuan Academy of Medical Science and Sichuan Provincial People’s Hospital Research Ethics Committee, and all trials were carried out in accordance with the necessary standards and laws.

The thyroid function profile, which comprises TT3, TT4, fT3, fT4, TSH, TgAb, and TPOAb (thyroid peroxidase antibody), was assessed in 526 euthyroid patients with valvular heart disease who were hospitalized for cardiac surgery department from January 2019 to June 2021. At the same time, data on 155 healthy individuals’ thyroid function and demographics, such as their age and gender, were gathered from the Sichuan Provincial People’s Hospital’s Health Examination Center. The primary first exclusion requirements were: 1) A TPOAb level of greater than 30 IU/ml or a TgAb level of greater than 75 IU/ml; 2) A history of thyroid disease; 3) A history of thyroid-related drug use, including lithium, amiodarone, interferon-alpha, interleukin-2, tyrosine kinase inhibitors, phenytoin, phenobarbiturates, iodine contrast, and carbamazepine; and 4) A history of pituitary disease.

### Clinical data collection

Patients with valvular heart disease demographic data were gathered, including their age, sex, weight, height, smoking, alcohol consumption, history of chronic illness, and drug use. Heart rate, blood pressure, pulse rate, and respiration rate were recorded as baseline clinical features. On the first day of admission, blood samples were taken and examined concurrently. Lymphocyte count, CRP, alanine transaminase, aspartate aminotransferase, total bilirubin, direct bilirubin, glucose, creatinine, blood urea nitrogen, cardiac troponin I, creatine kinase (CK), creatine kinase (CK), creatine kinase (CK), creatine kinase (CK), and creatine kinase, muscle and brain (CK-MB). The level of heart failure was also determined by looking at the brain natriuretic peptide (BNP). Heart surgery indicators such as cardiopulmonary bypass time (CPBT) and aortic occlusion time were gathered (AOT). Patients recovering from heart surgery in our cardiac surgery center are frequently transferred to the intensive care unit (ICU) for post-operative observation. For the severity assessment of the post-cardiac surgery patients, we additionally recorded ICU characteristics such as the sequential organ failure assessment (SOFA) score and acute physiology and chronic health illness categorization system II (APACHE II). The use of invasive and non-invasive ventilators, endotracheal intubation time, the likelihood of re-tracheal intubation, lung infections, intra-aortic balloon pumps (IABP), continuous renal replacement therapy (CRRT), extracorporeal membrane oxygenation (ECMO), and ICU time was also noted. After being released, the outcome of the hospital stay and deaths were also recorded. During or after data collection, none of the authors had access to information that may identify specific participants.

As patients were admitted, doctors and patients graded the NYHA class (New York Heart Association functional classification) scores following the NYHA functional classification (15).

### Thyroid hormone sampling

An automated chemiluminescence assay (Immulite 2000sr; Abbott, Shanghai, China) was used to evaluate the thyroid function profile, including TT3, TT4, fT3, fT4, TSH, TgAb, and TPOAb, immediately after sampling at the central laboratory of the Sichuan Provincial People’s hospital under routine external quality control. Based on data showing an analytical sensitivity of 0.0025 IU/ml for TSH, 1.5 pg/ml for fT3, and 0.22 ng/dl for fT4, the test was given regulatory approval. The intra- and inter-assay coefficients of variance were 5% and 10%, respectively, across all assays. TSH 0.35-4.94 mIU/l, fT3 2.43-6.01 pmol/l, fT4 9.01-19.05 pmol/l, TT3 (0.88-2.44nmol/L), and TT4 (62.68-150.8nmol/L) are the reference ranges used in our lab. TPOAb has a 30 IU/mL upper limit, while TgAb has a 75 IU/mL upper limit.

The presence of both a normal serum TSH level (0.35–4.94 mIU/L) and a serum T3 level below the lowest laboratory normal limit (0.88nmol/L) was used to diagnose ESS (16).

### Statistical analysis

Statistical evaluations were carried out utilizing SPSS version 23. (SPSS Inc., Chicago, IL, USA). Statistical significance was set at p 0.05 for all two-sided analyses. Depending on the situation, data are reported as mean ± SD (standard deviation), median (quartile range), or n(%). ANOVA and the student’s t-test were used for normally distributed variables, and the Mann-Whitney U and Kruskal-Wallis tests were used for nonparametric variables. Bonferroni’s *post hoc* comparison procedure, Dunnett’s unequal variance procedure, and Kruskal-Wallis one-way ANOVA were employed, respectively, for *post hoc* comparisons. For categorical variables, the Chi-squared test and Fisher exact probability approach were employed. In parametric statistical analysis, log-transformed was used for variables with known lognormal distributions (such as TSH and BNP). To investigate the correlations between the thyroid function and continuous variables, Pearson or Spearman rank correlation analysis and linear regression analysis were carried out. Analysis of covariance was used to examine how various effect modifiers interacted with one another. Also, the extent to which age influences the association between thyroid function and the clinical features of valvular heart disease was investigated using hierarchical analysis.

## Results

### Characteristics of thyroid function in patients with valvular heart disease

#### Differences in thyroid function between euthyroid patients with valvular heart disease and normal population

**Table 1** summarizes the sex, age, and thyroid function data for euthyroid patients with valvular heart disease and the general population. There was no clinically meaningful difference in TSH levels between the two patient groups (p>0.05). However, euthyroid individuals with valvular heart disease had lower levels of fT3, TT3, and TT4 than healthy patients (4.3 vs. 4.63; 1.37 vs. 1.48; 97.7 vs. 102.09, respectively; all p<0.05). On the other hand, patients with euthyroid valvular heart disease had higher fT4 levels (12.91 vs. 12.35, p<0.05).

**Table 1:** Differences in thyroid function between patients with valvular heart disease and normal population.

	Healthy control	VHD patients	P value
**Number**	155	526	
**Male**	76 (49%)	242 (46%)	
**Age**	49 ± 12	52 ± 12.5	
**fT3**	4.63 ± 0.6	4.3 ± 0.8	0.000*
**fT4**	12.35 (11.61-13.03)	12.91 (11.90-14.22)	0.000†
**TT3**	1.48 ± 0.2	1.37 ± 0.28	0.000*
**TT4**	102.09 ± 16.7	97.7 ± 19	0.000*
**TSH**	1.88 ± 1.8	2.0 ± 2.0	0.32 ※*

The data are presented as the n(%), mean ± SD or median(interquartile range) unless otherwise specified. VHD, valvular heart disease; fT3, Free Triiodothyronine; fT4, Free Thyroxine; TT3, Total Triiodothyronine; TT4, Total Thyroxine; TSH, Thyroid stimulation Hormone. Unit: fT3(pmol/L), fT4(pmol/L), TT3(nmol/L), TT4(nmol/L), TSH(mIU/L).

*Student`s t test was employed for normal distributed variables.

†The Mann-Whitney U test was employed for asymmetrically distributed variables.

※Log transform was use in parametric statistical analyses.

#### Differences in thyroid function according to NYHA grades in euthyroid patients with valvular heart disease and normal population

**Figure 1:**
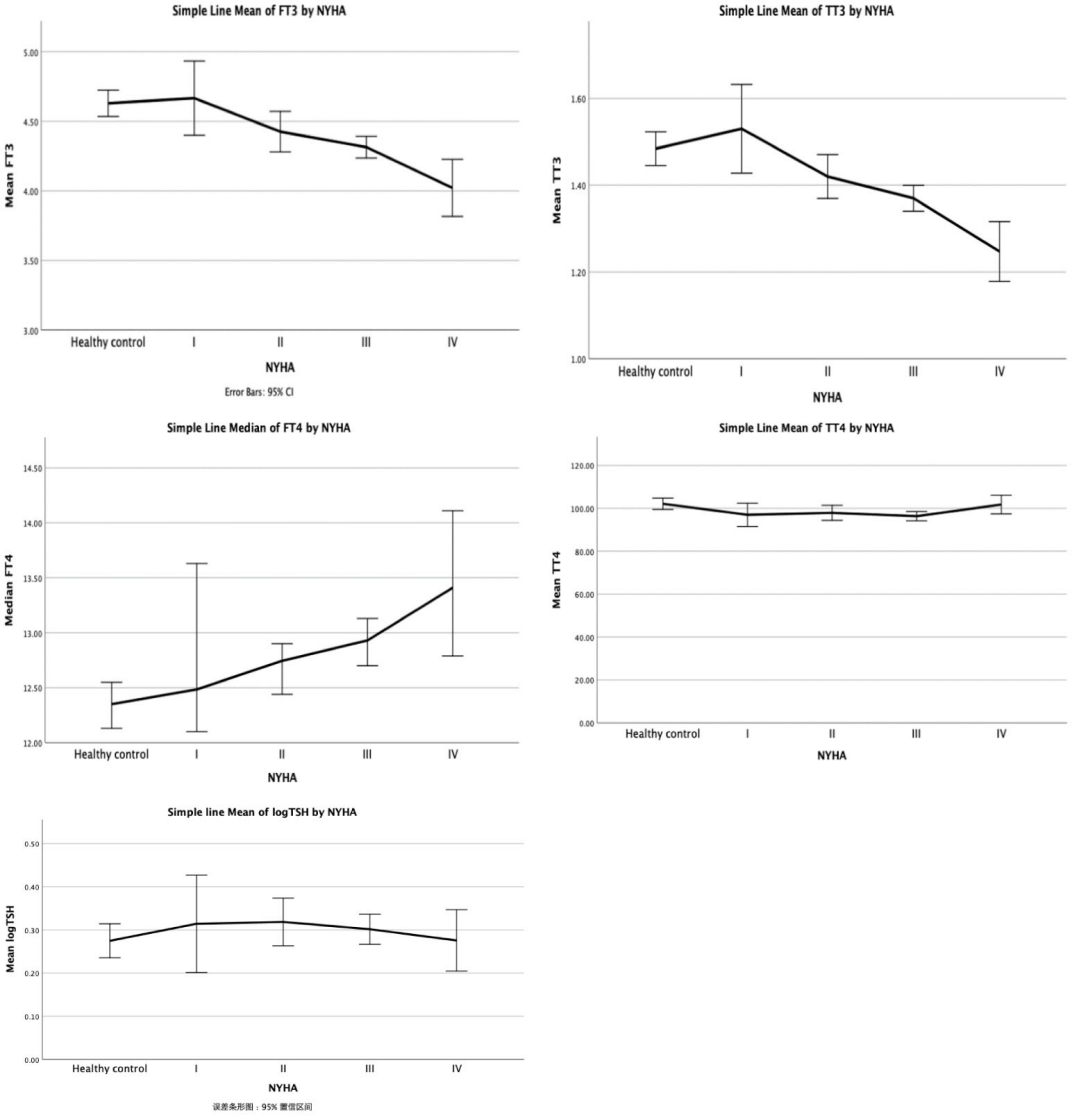
Thyroid function in patients with different NYHA grades of VHD.

To further explore the connection between thyroid function and heart function, we divided the VHD patients into subgroups using the NYHA classification (**Table 2**). ANOVA analysis revealed that there were statistically significant differences in the levels of fT3, fT4, TT3, and TT4 between the groups (all p<0.05), except for the TSH level. In particular, the levels of fT3, fT4, and TT3 all showed decreased trends in the various NYHA groups (**Figure 1**), but the level of TT4 did not show a clear trend. The subsequent hoc study revealed that there was no difference in thyroid function between valvular heart disease of NYHA grades 1 and 2 and healthy controls, but that there was a significant difference between the thyroid functions of valvular heart disease of NYHA grades 3 and 4 and healthy controls.

**Table 2:** Differences in thyroid function according to NYHA class in patients with valvular heart disease and normal population.

		VHD(NYHA class)				
	Healthy control	I level	II level	III level	IV level	P Value
**Number**	155	32	114	295	85	
**Male (%)**	76 (49)	20 (63)	47 (41.2)	140 (47.5)	35 (41.2)	
**age**	49 ± 12	39.7 ± 15.4	47.5 ± 13.7	54.1 ± 10.5	54.4 ± 11.85	
**fT3**	4.63 ± 0.6^3,4^	4.7 ± 0.74^4^	4.43 ± 0.78^4^	4.31 ± 0.68^0^	4.02 ± 0.95^0,1,2^	0.000*
**fT4**	12.4 (11.6,13.0)^3,4^	12.5 (12,14)	12.7 (11.9,13.9)^4^	12.9 (11.8,14.2)^0^	13.4 (12.2,15.2)^0,2^	0.000†
**TT3**	1.48 ± 0.2^3,4^	1.53 ± 0.28^3,4^	1.42 ± 0.27^4^	1.37 ± 0.26^0,1,4^	1.25 ± 0.32^0,1,2,3^	0.000*
**TT4**	102.09 ± 16.7^3^	96.98 ± 15.1	97.88 ± 18.8	94.3 ± 18.8^0^	101.7 ± 20.2	0.008*
**TSH**	1.88 ± 1.8	2.01 ± 2.01	2.08 ± 1.98	2.0 ± 2.0	1.89 ± 2.14	0.720※*

The data are presented as the n (%), mean ± SD or median (interquartile range) unless otherwise specified. VHD, valvular heart disease; fT3, Free Triiodothyronine; fT4, Free Thyroxine; TT3, Total Triiodothyronine; TT4, Total Thyroxine; TSH, Thyroid stimulation Hormone. Unit: fT3(pmol/L), fT4(pmol/L), TT3(nmol/L), TT4(nmol/L), TSH(mIU/L).

*ANOVA test was employed for normal distributed variables. Post hoc comparisons were made using Bonferroni’s method for equal variances, and Dunnett’s method was used for unequal variances.

†The Kruskal-Wallis tests test was employed for asymmetrically distributed variables, and one-way ANOVA for post hoc comparison.

※Log transform was use in parametric statistical analyses.

Number: 0-4 respectively represent healthy control, I level, II level, III level, IV level, and superscript number means statistically significant with relevant group in post hoc comparisons.

#### The incidence of ESS in different NYHA grades in euthyroid patients with valvular heart disease

Meanwhile, we compared several cardiac functions to describe the prevalence of ESS in valvular heart disease (**Table 3**). Patients with valvular heart disease and NYHA 1-3 grades did not have a substantially different incidence of ESS (all p>0.05); however, patients with NYHA 4 grades had a significantly greater incidence of ESS (16%, p<0.05).

**Table 3:** Incidence of euthyroid sick syndrome in patients with valvular heart disease by different NYHA classes.

NYHA class	VHD				
	I level	II level	III level	IV level	P Value
**Total number**	32	114	295	85	
**Euthyroid sick syndrome**	2	5	22	14*	0.016
**Percentage (%)**	6.25	4.39	7.46	16.47	

The Chi-squared test was used for categorical variables.

* Statistically significant with other groups in post hoc comparisons.

Influencing factors of thyroid function in euthyroid patients with valvular heart disease

**Figure 2:**
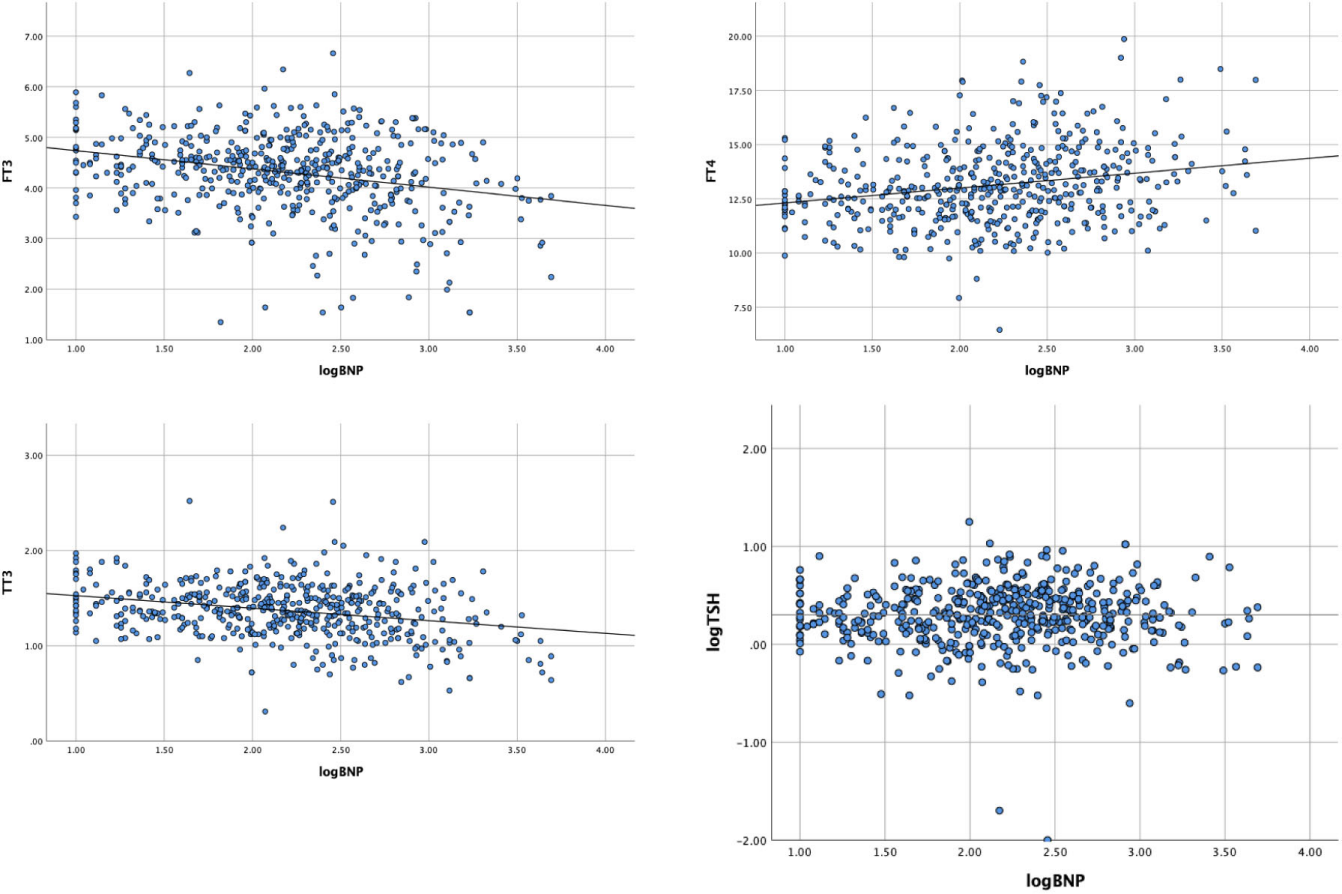
Simple scatter of thyroid function index by log BNP.

Age, sex, body mass index (BMI), and β-blocker therapy were investigated as variables impacting thyroid function in euthyroid patients with valvular heart disease. There was no gender-related difference in thyroid function (p>0.05). The findings indicated that older age was related to lower TSH levels (r -0.101, p <0.05). The relationship between fT3 and TT3 levels and BMI was found to be minimal (p< 0.05). Lower fT3 and TT3 levels were discovered to be related to treatment with β-blockers (r -0.135, p0.05; r -0.063, p<0.05, respectively). fT3 decreased by 30%, fT4 increased by 83%, and TT3 decreased by 12% for every 10-fold rise in BNP level, demonstrating the close relationship between thyroid function and BNP (**Table 4** and **Figure 2**).

**Table 4:** Effect of BNP on thyroid function.

BNP	Regression coefficient	Crude OR	Adjusted OR*
**fT3**	-0.361 (-0.47- -0.25)	0.67 (0.63-0.78)	0.70 (0.62-0.78)
**fT4**	0.685 (0.428 - 0.942)	1.98 (1.53-2.57)	1.83 (1.41-2.38)
**TT3**	-0.132 (-0.172 - -0.092)	0.88 (0.84-0.91)	0.88 (0.84-1.09)

* Adjusted for BMI (body mass index) and usage of β-blocker.

### Relationships between fT4 level and clinical characteristics of valvular heart disease

**Figure 3:**
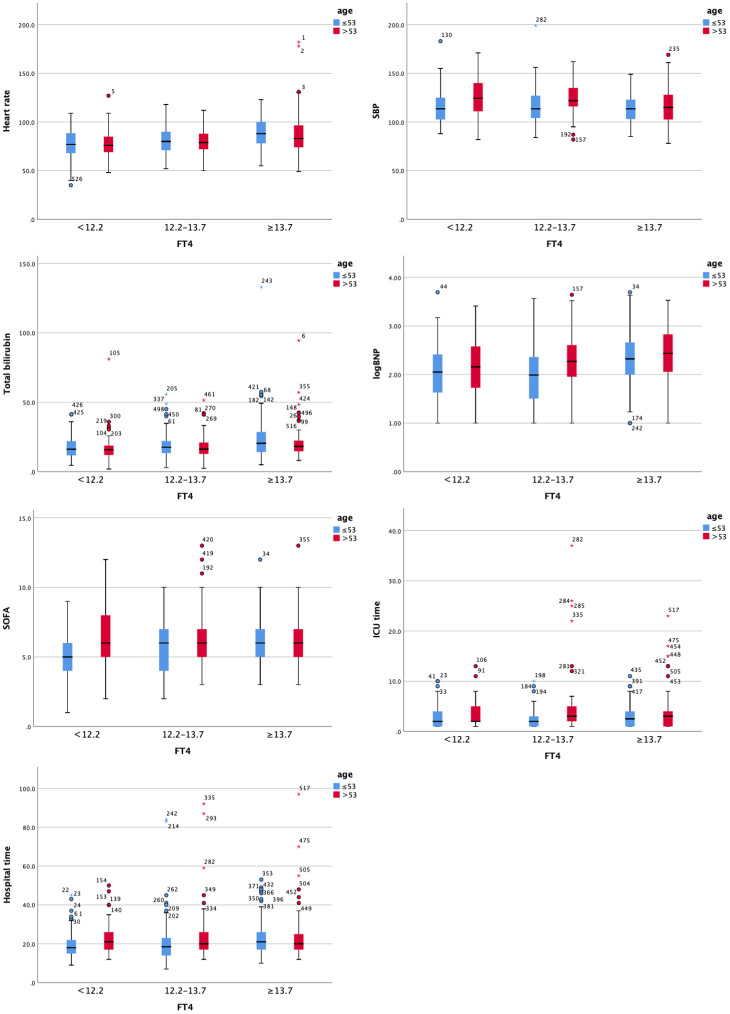
Clustered Boxplot of clinical index by fT3. The * represents outliers, which is a way of visualizing them in a box plot.

To conduct a hierarchical analysis, we divided all patients with valvular heart disease into three equal groups based on fT4 levels, using 53 as the age cutoff (**Table 5** and **Figure 3**). In the subgroup of patients under the age of 53, higher fT4 levels were associated with increased heart rate, elevated diastolic BP, higher total bilirubin, higher direct bilirubin, and elevated BNP levels (all p<0.05) upon admission to the hospital. Simultaneously, as fT4 levels increased, the SOFA score gradually increased in the ICU (p<0.05). In terms of outcome measures, increased F4 levels were closely correlated with longer ICU stay and overall length of stay (all p<0.05). Although in the subgroup of patients above the age of 53, higher fT4 levels were associated with an increased prevalence of hypertension, heart rate, systolic blood pressure, AST, total bilirubin, and BNP levels (all p<0.05), there was no strong association between higher fT4 levels and postoperative outcome measures in VHD patients. Although there was a statistical difference in intubation time, no increasing trend in intubation time was observed with increasing fT4 levels.

**Table 5:** Relationships between FT4 and clinical characteristics of patients with VHD at different ages.

Age (year)		≤53					>53				
fT4 Level(pmol/L)		ALL	<12.2	≥12.2 <13.7	≥13.7	P	ALL	<12.2	≥12.2 <13.7	≥13.7	P
**Number**		283	88	102	93		242	90	69	83	
**Baseline Characteristics**	BMI (kg/m^2^)	23 (3)	23 (3)	23 (4)	23 (3)		23 (4)	23 (3)	24 (4)	24 (4)	
	Hypertension	25	10	9	6		65	30	12	23	p<0.05
	Heart rate	82 (16)	77 (15)^3^	82 (14)^3^	88 (16)^1,2^	p<0.05	82 (17)	78 (13)^3^	80 (12)^3^	87 (22)^1,2^	p<0.05
	Systolic BP	116 (16)	116 (17)	117 (17)	114 (14)		122 (19)	125 (19)^3^	124 (18)^3^	116 (18)^1,2^	p<0.05
	Diastolic BP	69 (12)	67 (13)^23^	70 (11)^1^	71 (13)^1^	p<0.05	70 (15)	70 (17)	69 (12)	72 (14)	
	Lymphocyte	1.7 (0.6)	1.7 (0.5)	1.7 (0.6)	1.6 (0.7)		1.5 (0.5)	1.5 (0.5)^3^	1.6 (0.5)	1.5 (0.6)^1^	
	ALT(U/L)	24 (15-40)	21 (15-33)	24 (15-35)	28 (16-53)		23 (16-38)	22 (15-35)	21 (14-35)	26 (18-45)	
	AST(U/L)	41 (102)	34 (42)	45 (145)	43 (86)		32 (18)	30 (12)^3^	28 (11)^3^	38 (25)^1,2^	p<0.05
	TBIL (μmol/L)	18 (13-24)	16 (12-22)	18 (13-22)	20 (14-29)	p<0.05	17 (13-21)	16 (12-19)	16 (13-22)	18 (15-23)	p<0.05
	DBIL (μmol/L)	5.7 (4-8)	5.3 (4-7)	5.4 (4-7)	6.6 (4.9-8.4)	p<0.05	5.4 (4.4-7.2)	5.0 (3.9-6.5)	5.4 (4.3-7.0)	6.2 (5.0-8.4)	
	Glucose(mmol/L)	5.2 (2.9)	5.1 (1.0)	5.4 (4.5)	5.2 (1.3)		5.8 (2.1)	5.6 (2.0)	5.8 (1.6)	6.0 (2.7)	
	BNP (pg/mL) *	124 ± 4.0	,105 ± 4.0^3^	91 ± 3.8^3^	201 ± 3.7^1,3^	p<0.05	194 ± 3.8	138 ± 3.8^3^	204 ± 3.4	270 ± 3.9^1^	p<0.05
**Surgery related Indicators**	CPBT (min)	127 (50)	131 (59)^3^	125 (52)	125 (39)^1^		138 (52)	132 (50)	140 (56)	141 (50)	
	AOT (min)	86 (40)	86 (43)^3^	84 (40)	88 (36)^1^		92 (36)	89 (37)	91 (35)	97 (36)	
**ICU related indicators**	SOFA	5.8 (1.8)	5.4 (1.6)^3^	5.9 (1.8)	6.1 (1.9)^1^	p<0.05	6.2 (1.9)	6.5 (1.8)	6.3 (1.9)	5.9 (1.8)	
	APCHE II	10 (3.4)	11 (4.0)	10 (3.0)	10 (3.3)		12 (4.0)	12 (3.2)	12 (4.0)	11 (4.9)	
	Intubation time (hr.)	26 (45)	34 (69)	21 (30)	23 (25)		28 (40)	21 (19)^3^	38 (60)^3^	28 (34)^1,2^	p<0.05
	Re-tracheal intubation	5	1	1	3		11	3	5	3	
	IABP	14	6	1	7		14	6	4	4	
	CRRT	8	1	3	4		11	2	4	5	
	ICU time (day)	2 (1-4)	2 (1-4)	1 (1-3)	3 (1-4)	p<0.05	3 (1-5)	2 (2-5)	3 (2-5)	3 (1-4)	
**Outcomes**	Hospitalization time (day)	19 (15-24)	18 (15-22)	19 (14-23)	21 (17-26)	p<0.05	20 (17-26)	21 (17-26)	20 (17-27)	20 (17-25)	
	Death	3	0	2	1		13	4	5	4	

The data are presented as the n (%), mean ± SD or median (interquartile range) unless otherwise specified. VHD, valvular heart disease; fT3, Free Triiodothyronine; BP, blood pressure(mmHg); ALT, alanine transaminase; AST, aspartate aminotransferase; TBIL, total bilirubin; DBIL, Direct Bilirubin; BNP, brain natriuretic peptide; CPBT, cardiopulmonary bypass time; AOT, aortic occlusion time; SOFA, sequential organ failure assessment score; APCHE II, acute physiology and chronic health disease classification system II; IABP, intra-aortic balloon pump; CRRT, continuous renal replacement therapy.

ANOVA test was employed for normal distributed variables. Post hoc comparisons were made using Bonferroni’s method for equal variances, and Dunnett’s method was used for unequal variances.

The Kruskal-Wallis tests test was employed for asymmetrically distributed variables.

The Chi-squared test was used for categorical variables.

*Log transform was use in parametric statistical analyses.

Number: 1-3 respectively represent tripartite grouping of fT3, and superscript number means statistically significant with relevant group in post hoc comparisons.

p<0.05 was defined as statistical significance.

## Discussion

Our study revealed that, even with acceptable cardiac function, with an increase in NYHA grade, euthyroid patients with valvular heart disease exhibited a continuous trend of decreased fT3, TT3, and TT4 levels and increased fT4 levels compared to healthy people, except for TSH levels, which is inconsistent with the typical profile of patients with the ESS. Further investigation revealed that even after adjusting for other contributing factors (such as BMI and use of β-blockers), there was an 83% increase in fT4, a 30% decrease in fT3, and a 12% decrease in TT3 for every 10-fold increase in BNP. Also, other clinical signs are worse when fT3 levels are altered, but age is a significant influencing factor. Higher fT4 levels, higher heart rate, increased heart rate, elevated diastolic BP, higher total bilirubin, higher direct bilirubin, and elevated BNP levels, higher SOFA scores, are associated with longer ICU stay and overall length of stay in individuals under the age of 53 years. Many of the connections; however, were not present in patients over the age of 53 years.

The present study was a significant retrospective inquiry that targeted individuals with valvular heart disease who did not have any other metabolic disorders other coronary heart disease (17, 18). To minimize the effect on thyroid function, we also eliminated patients with thyroid illnesses (9, 19–21). In addition to examining thyroid function in patients with valvular heart disease, we also investigated how thyroid function related to other clinical indicators, such as baseline characteristics, indicators related to surgery and the intensive care unit, as well as outcome measures such as hospital stay and death. In euthyroid individuals with valvular heart disease, our study provides the first comprehensive description of the thyroid profile, including TSH, fT3, fT4, TT3, and TT4, with specific attention to the various NYHA classes and their relationships to clinical indicators.

The ESS has been implicated in numerous earlier investigations as the cause of aberrant thyroid functioning in patients with cardiac insufficiency (22). In patients with either acute or chronic systemic diseases, the ESS is characterized by a typical laboratory values, notably low T3 levels with normal or low T4 and TSH levels, in patients with acute or chronic systemic diseases (23). In contrast to previous trials, our study revealed that the incidence of ESS was, 6.25%, 4.39%, and 7.46% in patients with NYHA classes I through III, respectively. The incidence of ESS did not differ statistically across the three groups. However, there was a downward trend observed in fT3 and TT3 levels among the valvular heart disease population compared to the healthy population. It is noteworthy that fT3 and TT3 levels may be affected in patients with NYHA I-III levels, even though the incidence of the ESS is not higher than that of the healthy population. While the ESS may contribute to these findings, individuals with valvular heart disease did exhibit relatively low T3 levels, and higher T4 levels, particularly, fT4. The ranges of T3 and T4 levels observed are wider than what would typically be expected in euthyroid sick syndrome.

Traditionally, studies on thyroid function have primarily focused on TSH levels, which have been associated with a higher risk of heart failure, changes in cardiac function, and even mortality. However, the CORONA experiment found no association between TSH levels and heart failure with a lower ejection fraction (3, 6, 7, 11, 24). Despite this, TSH has remained a crucial guiding signal for thyroid therapeutic intervention trials. In individuals with valvular heart disease, TSH levels, and T3 and T4 levels may not align consistently, potentially due to abnormal conversion of T4 to T3 in peripheral tissues (20, 25, 26). Importantly, T3 or T4 levels may have greater clinical significance in these patients. Therefore, in certain trials, particularly those involving patients with abnormal cardiac function, TSH, T3, and T4 levels should be considered, and T3 and T4 levels could potentially be used as a management target.

Additionally, to the changes in T3 levels, patients with valvular heart diseases also experienced different changes in fT4 levels when compared to healthy individuals and the fT4 level increased with the increase of NYHA grade and BNP level. While T3 is recognized as the active cellular form of thyroid hormones, it`s important not to underestimate the role of T4. The Penn Heart Failure Study found that atrial fibrillation was positively associated with higher levels not fT3 or TT3, meanwhile, higher fT4 levels were also linked to incident heart failure, and sudden cardiac death (9, 13, 14). Although our cross-sectional study does not establish a cause-and-effect relationship, the close relationship between fT4 levels and NYHA levels, and BNP levels further underscores the crucial role of fT4 levels in patients with cardiac insufficiency. The peripheral deiodination of T4 to T3 may be affected by an increase in cytokines, free fatty acids, and cortisol, resulting in higher fT4 levels that reflect the worsening condition of heart failure.

The analysis of thyroid function and clinical features yielded intriguing results, indicating a connection between fT4 levels and several clinical parameters, with age playing a significant role in this association. The strength of the association between fT4 levels and clinical characteristics appears to be more pronounced in younger patients, while it is less prominent in older patients. This could be attributed to the fact that cardiac performance and clinical indicators in older patients are influenced by multiple uncorrected factors, making it more challenging to achieve favorable outcomes. These findings suggest the potential use of certain interventions, such as administering thyroxine to younger patients, to improve clinical outcomes. By targeting fT4 levels and addressing thyroid function in younger patients, it may be possible to enhance their overall prognosis.

The absence of the rT3 level due to technical limitations in our center’s laboratory is the study’s limitation; however, this does not affect our analysis of the results, and in addition, most laboratories are unable to detect the level of rT3, so there are some restrictions on its potential clinical applications. At the same time, it was impossible to completely rule out the potential of central hypothyroidism. The second drawback is that patient mortality and death outcomes cannot be studied due to the low mortality rate before discharge and the short follow-up period. Monitoring thyroid function at multiple time points may provide a better understanding of the changes in thyroid hormone levels throughout the process. However, due to the retrospective design of the study, this may be considered a limitation of the current trial. Meanwhile, due to limited resources, we were unable to obtain sufficient imaging-based indicators before and after cardiac surgery, which may partially limit the scope of our study. In the future, more research can be done to get beyond these restrictions.

## Conclusions

In conclusion, our study showed that euthyroid patients with valvular heart diseases had decreased fT3, TT3, and TT4 levels compared to healthy individuals, but increased levels of fT4 even with acceptable cardiac function. Additionally, they showed a continuous trend with the change in NYHA grade, which is inconsistent with the profile of ESS. Further research showed that for every 10-fold rise in BNP, fT4 increases by 83%, fT3 decreases by 30%, and TT3 lowers by 12%, even after accounting for other influencing variables. Additionally, there are various clinical indications that are connected to the adjusted level of fT4 that are associated with worse outcomes; however, age is a crucial influencing factor since many of the associations between the adjusted level of fT3 and clinical indicators are only evident in individuals under the age of 53. As a result, not only TSH but also T3, and T4 levels should be of concern in some trials, especially in patients with abnormal cardiac function, and T4 levels can even be used as a management target other than TSH. Further research is required to explore the use of thyroxine to improve clinical outcomes in abnormal thyroid function of valvular heart disease at a younger age.

## Data availability statement

The original contributions presented in the study are included in the article/**Supplementary Material**. Further inquiries can be directed to the corresponding author.

## Ethics statement

The studies involving human participants were reviewed and approved by Sichuan Academy of Medical Science and Sichuan Provincial People’s Hospital Research Ethics Committee. The patients/participants provided their written informed consent to participate in this study.

## Author contributions

The authors confirm their contribution to the paper as follows: study conception and design: PW, SW, SL,YW. Data collection: PW, SL, YY, LL. Analysis and interpretation of results: PW, SL, GZ. Draft manuscript preparation: PW, JZ, DN. All authors reviewed the results and approved the final version of the manuscript.
