# Supplementary material for: The severity of valvular heart disease in euthyroid individuals is associated with thyroid hormone levels but not with TSH levels
Source: Front Endocrinol (Lausanne). 2023 Jul 4;14:1193557. doi: 10.3389/fendo.2023.1193557 (PMC10352847; doi:10.3389/fendo.2023.1193557)
Supplement: Supplementary file 1 [file Table_1.docx]

Table 1. The influence of age on thyroid function

| Age | Spearman Rank Correlation | |
| --- | --- | --- |
|  | Correlation coefficient | P value |
| fT3 | -0.023 | 0.444 |
| fT4 | -0.003 | 0.941 |
| TT3 | -0.079 | 0.069 |
| TT4 | 0.013 | 0.764 |
| TSH | -0.101 | 0.021 |

Table 2. The influence of gender on thyroid function

|  | Man | Women | P value |
| --- | --- | --- | --- |
| Number | 242 | 284 |  |
| Age | 53(48-61) | 39(27-48) |  |
| fT3 | 4.3±0.8 | 4.5(3.9-4.8) | 0.314 |
| fT4 | 12.9(11.86-14.2) | 12.7(11.9-14.2) | 0.615 |
| TT3 | 1.37±0.3 | 1.4(1.2-1.5) | 0.531 |
| TT4 | 97.8±19.3 | 95.3(84.7-110.8) | 0.581 |
| TSH | 1.99±2.0 | 2.3(1,5-3.4) | 0.28 |

Table 3. The influence of BMI on thyroid function

| BMI | Pearson Rank Correlation | |
| --- | --- | --- |
|  | Correlation coefficient | P值 |
| fT3 | -0.135 | 0.002 |
| fT4 | -0.074 | 0.089* |
| TT3 | -0.063 | 0.031 |
| TT4 | -0.01 | 0.725 |
| TSH | -0.008 | 0.794 |

* Spearman rank correlation

Table 4. The influence of β blocker on thyroid function

|  | Non-β blocker | β blocker | P value |
| --- | --- | --- | --- |
| Number | 434 | 92 |  |
| Male | 204（47%） | 38（41.3%） |  |
| Age | 52(45-60) | 55(49-60) |  |
| fT3 | 4.3±0.8 | 4.2(3.9-4.6) | 0.021 |
| fT4 | 12.9(11.8-14) | 13.6(12.3-14.9) | 0.000 |
| TT3 | 1.38±0.3 | 1.3(1.1-1.5) | 0.015 |
| TT4 | 97.3±18.8 | 97.3(84.7-111.9) | 0.548 |
| TSH | 1.97±2.1 | 2.3(1.52-3.31) | 0.215 |

Table 5. The influence of BNP on thyroid function

| BNP^※^ | Pearson Rank correlation | |
| --- | --- | --- |
|  | Correlation coefficient | P value |
| fT3 | -0.278 | 0.000 |
| fT4 | 0.225 | 0.000* |
| TT3 | -0.277 | 0.000 |
| TT4 | 0.071 | 0.109 |
| TSH | 0.000 | 0.993 |

* Spearman rank correlation

※Log transform was use in parametric statistical analyses
